# Human Alpha Herpesviruses Infections (HSV1, HSV2, and VZV), Alzheimer’s Disease, and the Potential Benefits of Targeted Treatment or Vaccination—A Virological Perspective

**DOI:** 10.3390/vaccines13060572

**Published:** 2025-05-27

**Authors:** Peter A. C. Maple, Akram A. Hosseini

**Affiliations:** 1 Mental Health and Clinical Neuroscience Academic Unit, University of Nottingham School of Medicine, Nottingham NG7 2UH, UK; akram.hosseini1@nhs.net; 2 Department of Neurology, Nottingham University Hospitals NHS Trust, Queen’s Medical Centre, Nottingham NG7 2UH, UK

**Keywords:** Alzheimer’s disease, human alphaherpesviruses, herpes simplex viruses, varicella zoster virus

## Abstract

Understanding the contribution of human herpesviruses to the aetiology of neurodegenerative diseases is an emerging field of interest. The association of Epstein–Barr virus with multiple sclerosis is the most researched example; however, the definitive proof of causation is still lacking. Alzheimer’s disease (AD) is the most common form of dementia and typically manifests in individuals aged over 65 years; however, it also occurs in a small number of individuals aged less than 65 years. A combination of environmental, genetic, and lifestyle factors is believed to contribute to the development of AD. There have been several reports describing potential associations of infections or reactivations of human alphaherpesviruses with AD. A particular characteristic of human alphaherpesviruses (herpes simplex viruses 1 and 2, varicella zoster virus) is that they are neurotropic and that lifelong infection (latency) is established mainly in the dorsal root and trigeminal ganglia. There have also been reports that suppression of alphaherpesvirus infections through either vaccination or the application of antiviral treatments may be protective against the development of AD. Zoster vaccines and acyclovir may prove to be effective interventions for preventing or limiting the progression of AD. This is particularly relevant as there are currently no available cheap and effective treatments for AD. In this review, the basic virology of human alphaherpesviruses is described followed by their epidemiology and associations with AD. Finally, the prevention and treatment of human alphaherpesviruses are considered in the context of potential applications for the prevention of AD.

## 1. Introduction

Alzheimer’s disease (AD) is the most common form of dementia and typically manifests in individuals aged over 65 years; however, it also occurs in a small number of individuals aged less than 65 years. A combination of environmental, genetic, and lifestyle factors is believed to contribute to the development of AD [1,2]. Since the description of the defining pathology by Alzheimer and others approximately 120 years ago, the disease bearing his name has become a major global healthcare challenge [3]. According to the Global Burden of Disease Study 2016 [4], it has been estimated that between 1990 and 2016, the number of prevalent dementia cases more than doubled, increased from 20.2 million in 1990 to 43.8 million in 2016.

The objective of this review is to describe the associations between alphaherpesvirus and Alzheimer’s disease. This will be performed from a virological perspective. Finally, potential virological interventions (e.g., antiviral treatment and vaccination) will be explored.

## 2. The Human Alphaherpesviruses

Herpesviruses are double-stranded DNA viruses characterized by a typical virological structure comprising an icosahedral capsid surrounded by a tegument packaged within a lipid envelope containing glycoproteins [5]. Taxonomically [6], the family *Herpesviridae* includes three subfamilies (*Alphaherpesvirinae*, *Betaherpesvirinae*, and *Gammaherpesvirinae*) comprising over 10 genera and more than 100 species. The host range includes mammals, birds, and reptiles. Nine species of herpesviruses are recognized to infect humans. They share the capacity to develop latency [7] following primary infection, in which they enter a non-replicative state but retain the capacity to reactivate, particularly if the host’s immune control is diminished. The human alphaherpesviruses (Table 1), herpes simplex virus 1 (HSV1), herpes simplex virus 2 (HSV2), and varicella zoster virus (VZV) establish latency in neurons [8]. These viruses are neurotropic and infection, or reactivation can directly result in severe neurological disease, e.g., encephalitis/meningitis [9,10]. There is also growing awareness that alphaherpesvirus infection may be associated with, or impact upon, several neurodegenerative diseases through mechanisms which remain to be determined [11,12,13].

## 3. The Herpes Simplex Viruses

### 3.1. Virology

Descriptions of herpetic infections can be traced back to antiquity; however, Richard Morton is credited with giving the first specific description of herpes febrilis (cold sores) in 1694 [14]. Herpes genitalis (genital herpes) was first specifically described by Jean Astruc in 1736 [15]. Subsequently, the term Herpes Simplex has been used to embrace several clinical manifestations of herpetic infection (e.g., febrilis, genitalis, labialis, and zoster). Early in the 20th century, it was established that a virus was responsible for Herpes Simplex, and from the 1960s, several clinical, epidemiological, and laboratory studies have shown that there are two distinct herpes simplex viruses [16,17,18]. Herpes simplex virus 1 (HSV1) and herpes simplex virus 2 (HSV2) genomic DNA sequences were published in 1988 [19] and 1998 [20], respectively. The DNA sequences of HSV1 and HSV2 are closely aligned, comprise 152,261 and 154,746 base pairs, respectively, and show close amino acid sequence homology [21]. Initially, their close antigenic similarity made it difficult to serologically differentiate between the two viruses [22]; however, by the mid-1980s, the type-specific glycoprotein gG had been identified [23,24]. Serological assays using either glycoproteins gG-1 (HSV1) or gG-2 (HSV2) have become an accepted standard for the measurement of HSV-specific antibodies [25,26] and, therefore, underpin HSV association studies based on the detection of HSV1- and/or HSV2-specific antibodies. It is important to note that these assays do have limitations that include variable sensitivities and specificities in detecting primary or recurrent infection [27], inaccuracies [28], and low specificity for HSV2 IgG in African populations [29]. Finally, gG antibody assays are designed to differentiate HSV1 from HSV2 based upon the detection of a targeted antibody, but such assays may be inappropriate for association studies in which detection of a more diverse range of antibodies is desirable.

Herpes simplex viruses establish latency following primary infection, and their disease manifestations reflect this property. Typically, primary infection commences with virus invasion of cells of the mucosal epithelia followed by direct entry into axons and subsequent transport to nuclei of the sensory ganglia where latency is established [30]. Periodically, the virus can reactivate to a lytic/replicative state, and new viruses then undertake the reverse journey to the periphery. Many people experience HSV infection during their lives as a clinically insignificant or self-resolving event [31]; however, in certain groups, HSV infection or reactivation can lead to severe complications. Neonatal herpes [32] is a medical emergency as is herpes simplex encephalitis [33]; both diseases are responsible for significant mortality and long-term neurological sequelae. Genital herpes [34] is widespread, commonly undiagnosed, a source of stigma, and associated with an increased risk of HIV transmission. Finally, HSV reactivation in immunocompromised patients can have several serious manifestations including oesophagitis, meningoencephalitis, pneumonitis, hepatitis, and pneumonitis [35].

### 3.2. Epidemiology

The development of serological methods to differentiate HSV1 from HSV2 has enabled reliable seroepidemiological data to be collected for these two viruses. Early studies [36], which were much focused on genital herpes, reported varying seroprevalences associated with age, sex, ethnicity, geography, lifestyle factors, and socioeconomic factors. For example, in a London-based study [37], 22.7% of patients attending a genitourinary medicine department were HSV-antibody-positive compared to a 7.6% seroprevalence in a control population of blood donors, strongly indicating an association between HSV2 infection and sexual lifestyle. In another UK study [38], sera were collected from 3533 women attending antenatal clinics and tested for total HSV antibodies and HSV2-specific antibodies. The HSV1 seroprevalence was nearly 100% in women originally born in Africa or the Carribbean compared to 60–80% for those born in the UK. Furthermore, HSV2 seroprevalence ranged between 10% and 40%, depending upon the ethnic group sampled. Finally, much higher HSV2 seroprevalences (e.g., 70%) have been reported [39] from African countries compared to those from European countries, and HSV2 seroprevalences were significantly higher in HIV-infected individuals.

Recent studies [40] have shown that a significant proportion of Europe’s population is infected with HSV2 and that HSV2 infection accounts for approximately two-thirds of genital herpes cases. Similarly, in the Middle East and North African countries [41], the pooled mean HSV2 seroprevalence has been estimated at 5.1% with nearly three quarters of genital herpes cases attributed to HSV2 infection, and in the USA [42], the HSV2 seroprevalence has been estimated at 17.0%. The population rates of HSV-1 infection are considerably higher than those of HSV2 with a global prevalence of infection estimated at 66.6% in individuals aged 0–49 years [43]. In several regions, the historical epidemiology of HSV1 infection appears to be changing with increasing proportions of adolescents recorded as HSV1-susceptible and increasing rates of isolations from cases of genital herpes [42,44]. Typically, HSV1 infection rates peak during early childhood (e.g., prevalence of 30–60% in individuals aged over 1–5 years) and seroprevalence gradually increases during adolescence, although there is considerable variation across geographical locations [45]. HSV1 seroprevalence in adults can either gradually increase or plateau; however, there are marked variations, depending upon whether the population is categorized as “high risk” or “low risk” [46].

### 3.3. Epidemiological Associations with Alzheimer’s Disease (AD)

Early studies were conflicted regarding the potential role of HSV1 in the pathogenesis of Alzheimer’s disease [47,48,49]. Over time, increasing evidence has been presented, supporting an association of HSV1 infection and AD. In 1997, Itzhaki and colleagues [50] reported that the combination of HSV1 in brain and carriage of an APOE-ε4 allele is a strong risk factor for AD. Several studies [51] have reported that HSV nucleic acid is also detected in the brain of people without AD, and it has been proposed that HSV reactivation is linked to the development of AD. For instance, Mori and colleagues [52] based on molecular and in situ hybridization investigations provided the first evidence of HSV1 reactivation in people with familial AD. Furthermore, Letenneur and colleagues [53] in a prospective study of 512 elderly subjects reported HSV reactivation, as evidenced by HSV IgM seropositivity, was highly correlated with incident AD. HSV infection has been also reported [54] to be associated with AD when the host is coinfected with cytomegalovirus (another herpesvirus), and in a UK Biobank study [55], cumulative infection with four neurotropic herpesviruses significantly increased the risk of dementia. Table 2 summarizes several recent studies reporting an association between HSV infection and AD.

### 3.4. Prevention and Treatment

Several different types of herpes simplex vaccines (e.g., inactivated, live-attenuated, replication-defective, subunit, and modified RNA) have been developed; however, none have entered clinical use [61]. There is interest in the development of both prophylactic and therapeutic vaccines [62,63]. Historically, the focus of HSV vaccine development has been the prevention of genital herpes, and the application of HSV vaccines for the prevention or treatment of Alzheimer’s disease is subject to several caveats. Primarily, the role of HSV infection in the causation or progression of AD requires confirmation together with the biological mechanisms responsible. The application of antiviral agents to prevent or treat Alzheimer’s disease would appear to be a more realistic proposition at least in the short term. There are several antiviral agents available which are active against herpesviruses, and the acyclic guanosine analogue Acyclovir is a highly effective treatment for HSV infections [64,65]. Acyclovir has been shown to be a potent inhibitor of HSV replication and acts by inhibiting virus DNA polymerase following selective phosphorylation by virus thymidine kinases [66,67]. Treatment with acyclovir has been shown to reduce the duration of virus shedding in genital herpes (but not the frequency of recurrences) and reduce the morbidity and mortality associated with HSV encephalitis [68,69,70]. The use of acyclovir treatment as a potential way to prevent Alzheimer’s disease has been suggested [71,72,73]; however, to date, there has been very limited evidence in support of such an approach [74]. Determination of when to administer treatment and how long to undertake treatment, identification of markers of treatment success, and assessment of whether different patient groups (e.g., early onset disease versus late onset disease) require different approaches are all issues that need to be addressed. Furthermore, potential interactions between human herpesviruses, for example reactivation of HSV1 in the brain in response to VZV infection [75], also need to be considered, as well as the carriage of genetic risk factors (e.g., APOE-ε4 gene).

## 4. Varicella Zoster Virus

### 4.1. Virology

Primary infection with varicella zoster virus (VZV) is responsible for chickenpox (varicella), and reactivation is responsible for shingles (zoster). The clinical presentations of herpes-varicella and herpes-zoster and their potential relationship have been accurately described since the early 20th century [76,77] and have been extensively described elsewhere [78,79]. The confirmation that the same virus was responsible for both presentations was presented by TH Weller who first cultured the V-Z virus during the 1950s [80]. The varicella zoster virus is an enveloped, double-stranded DNA virus with a genome of approximately 125,000 base pairs comprising 68 unique genes [81]. Based on genome sequencing, several clades of the virus have been identified, which have varying geographical distributions [82]. For example, clade 2 viruses predominate in China [83], and viruses of clades 1 and 3 predominate in Europe [84].

VZV infection primarily occurs by the aerosol route, although skin vesicles which contain high titers of cell-free virus can also represent a source of transmission to others [85,86]. In aerosol transmission, VZV first establishes infection in the pharynx, and it is then disseminated throughout the body by circulating lymphocytes [87]. VZV preferentially infects T cells with skin homing properties [88], enabling the virus to initiate infection of the dermis and epidermis. Several studies [89] have shown VZV DNA to be present in T cells (viraemia) approximately 1–2 weeks prior to the appearance of the characteristic skin rash. Virus latency is established in human ganglionic neurons; however, the mechanisms by which the neurons become infected remain to be confirmed [90,91]. Herpes zoster is the typical manifestation of VZV reactivation, and the pathophysiology and clinical sequelae associated with this process have been described elsewhere [92,93].

Diagnoses of varicella and zoster are reliably achieved on the bases of clinical presentations in most cases [94,95], and molecular methods (e.g., PCR) can be used to confirm the presence of VZV DNA in vesicle fluid [96]. Both primary infection and VZV reactivation can lead to the central nervous system (CNS) infection with different presentations, e.g., encephalitis, meningitis, and myelitis. The CNS infection has also been associated with facial palsy. The detection of VZV DNA in CSF is the method of choice for the confirmation of these infections [97]; however, although highly specific, it lacks sensitivity beyond the initial acute phase [98]. Consequently, the detection of VZV intrathecal antibodies is recommended, particularly for PCR-negative cases [99]. Primary infection and reactivation by VZV in immunosuppressed individuals can lead to severe and potentially life-threatening infection. After bone marrow or solid-organ transplantation, reactivation of VZV is a significant problem as it can lead to severe disseminated infection and to the development of visceral zoster, which presents as severe abdominal pain and may be associated with the involvement of internal organs, such as the liver, colon, or lung. VZV reactivation has been shown to be a significant problem in patients with haematological malignancies or after haematopoietic stem cell transplantation.

Diagnosis of varicella by the detection of specific IgM is not generally recommended because of concerns about lower sensitivity compared to VZV DNA detection [100]. VZV IgG seroconversion or VZV IgM detection may be useful for confirming chickenpox in cases where the collection of vesicular fluid is not achievable. The sensitivity of detection of VZV IgM in patients with zoster has generally been reported to be low [101,102]; however, it has been suggested [103] that this can be improved by using class-specific antibody capture assays. Finally, a particularly difficult diagnostic challenge following VZV reactivation (e.g., in the enteric nervous system) is the diagnosis of zoster *sine herpete*, an atypical clinical manifestation of herpes zoster in which the characteristic rash is absent [104]. This condition which frequently manifests with severe pain is susceptible to misdiagnosis and represents a laboratory diagnostic challenge [105]. A combination of laboratory investigations is recommended [106,107], including testing for VZV DNA in blood/CSF, the demonstration of intrathecal VZV IgG, and the detection of VZV IgM.

### 4.2. Epidemiology

Historically, VZV infection has typically occurred in young children with most individuals showing immunity by their early teens. The rate of acquisition of infection varies considerably in different geographical regions and climates. The impacts of mass childhood varicella vaccination (MCVV) also need to be considered as different countries have adopted this strategy for varying lengths of time. In Western European countries [108], before the introduction of MCVV, VZV IgG seroprevalence in young children (<5 years) ranged between 35.3% (Greece) and 80.6% (The Netherlands), which increased to >90% by age 15 years in 14 of 16 countries. Universal vaccination of children aged 12–18 months using live attenuated varicella vaccine was introduced by the USA in 1996 [109]. Initially, one dose of vaccine was recommended; however, this was not sufficient to establish full protection [110], so a second dose was introduced in 2007 [111]. It has been widely shown that the antibody response following vaccination is not as robust as that following wild-type infection [112], and highly sensitive laboratory assays are recommended for its measurement [113,114]. The adoption of MCVV by the USA has led to a profound change in VZV epidemiology; for example, the nationwide average annual incidence of varicella declined 88.6% during the two-dose period from 28.7 per 100,000 population in 2005–2006 to 3.3 per 100,000 population in 2018–2019 [115].

It is well established that, in regions with a tropical climate, a significant number of adults remain susceptible to varicella [116,117]. For example, in Thailand, seroprevalence rates of 24.1% and 69.2% have been reported in the 1–4-year-old and 20–29-year-old age groups, respectively [118]. Similarly, in Singapore, VZV IgG seroprevalences of 34.5% in children aged 1–6 years and 84.0% in young adults aged 18–29 years have been reported [119]. In other regions, for instance Latin America [120], the rates of acquisition of VZV infection by age resemble those seen in European countries (pre-vaccine). Several hypotheses [121] have been put forward to explain the higher susceptibility of adults to VZV infection in tropical regions, including reduced transmission in rural populations versus urban populations and lower infectivity due to an increased exposure to ultra-violet radiation in tropical climates.

### 4.3. Epidemiological Associations with Alzheimer’s Disease

Several studies have reported associations of herpes zoster with Alzheimer’s disease [122]. Ukraintseva and colleagues [123] analyzed a pseudorandomized sample from the US Health and Retirement Study and concluded that shingles, pneumonia, and mycoses diagnosed in participants aged 65–75 were all associated with a significantly increased risk of AD later in life by 16–42%. Furthermore, pneumococcal and shingles vaccines administered between ages 65 and 75 were both associated with a significantly lower risk of AD by 15–21%. An association between herpes zoster and AD has been reported by Bae and colleagues [124], who analyzed the South Korean National Health Insurance-National Sample Cohort following individuals from 1 January 2002 to 31 December 2013. In this study, they reported an adjusted hazard ratio for AD following herpes zoster of 1.11 (95% CI: 1.04–1.19) and an overall adjusted hazard ratio for all dementias of 1.12 (95% CI: 1.05–1.19). Additionally, for patients who received antiviral therapy, there was a significantly lower risk of dementia. A further study of the South Korean National Health Insurance databank over the period between 2006 and 2017 has been undertaken by Shin and colleagues [125]. Following multivariate time-varying Cox regression analysis, VZV infection was shown to be associated with subsequent AD with a hazard ratio of 1.52 (95% CI: 1.46–1.57), and there was an increased risk of AD in individuals coinfected with HSV, with a hazard ratio of 1.75 (95% CI: 1.66–1.85). Finally, in a study of the Taiwan Longitudinal Health Insurance Database, Tsai and colleagues [126] identified an association of herpes zoster ophthalmicus with the subsequent risk of dementia. In their analysis, after adjusting for patients’ characteristics and comorbidities, herpes zoster ophthalmicus patients were at a 2.97-fold greater risk than controls for developing dementia (AD was not specifically identified). Despite the reported associations of herpes zoster with AD described in the aforementioned studies, two recent comprehensive meta-analyses [127,128] concluded that the evidence for the association of herpes zoster with dementia was equivocal with the caveat that additional studies (longitudinal with robust virus measurement and multi-centre) are needed. Considering the differences in VZV epidemiology between temperate and tropical climates, there is a particular need for studies of herpes zoster associations with AD conducted in populations living in temperate climates.

### 4.4. Prevention and Treatment

Several antivirals are available for the treatment of VZV infections/reactivations (Table 3), together with varicella and zoster vaccines.

Theoretically, VZV reactivation may play a role in neurodegenerative processes. Thus, preventing VZV reactivation could potentially mitigate or slow down the progression of neurodegeneration triggered by such reactivation. Acyclovir, valaciclovir, and famciclovir—antiviral agents that have been shown to reduce the severity and duration of VZV reactivation—could indirectly benefit AD patients by potentially reducing neuroinflammation associated with viral reactivation. In registry-based or population-based studies [124,139], antiviral treatment of herpes infections with antivirals reduced the long-term risk of subsequent dementia. 

Zoster vaccines [140,141] may help reduce the likelihood of inflammation or viral-induced neurodegeneration by preventing VZV reactivation. Several studies [142,143,144,145] have reported that zoster vaccination appears to reduce the risk of developing dementia (Table 4). Recently, Eyting and colleagues [143] have published the results of a natural experiment on the effect of herpes zoster vaccination on dementia. In this study, they provided evidence of a dementia-preventing or dementia-delaying effect from zoster vaccination that is less vulnerable to confounding and bias compared to other associated studies. Furthermore, the recombinant zoster vaccine [144] may be associated with lower risks of dementia compared with two other vaccines commonly used in older adults including influenza and tetanus. In addition, it has been suggested [145] that zoster vaccination of younger age groups should be considered as a means to reduce the risk of developing dementia. It remains unclear whether different zoster vaccines types including zoster mRNA vaccines in development have varying preventive effects [146,147].

Reviews and clinical studies have explored the potential of BCG vaccination in reducing herpes recurrences, with evidence from a nested clinical trial showing a reduced risk of recurrence for up to six months [148,149,150]. One study by Hippman and colleagues [151] showed that a single BCG injection significantly reduced herpes labialis recurrences in patients, with many experiencing no recurrences for 4–6 months and some for up to 10 years. Although the effect diminished over time, a small percentage of patients remained recurrence-free for over 3 years. Given the potential link between preventing HSV reactivation and reducing Alzheimer’s disease risk, further research into BCG’s role is warranted.

There are conflicting reports [152,153] on APOE genotypes conferring risk of reactivation of VZV (shingles). Two studies examined the combined risk of Alzheimer’s disease linked to HSV1 and the APOE-ε4 gene. Lindman et al. [154] have found that individuals with one APOE-ε4 gene and antibodies against HSV1 had a higher risk of developing AD. This risk was much greater for those with two APOE-ε4 genes, but there was no increased risk for people without the APOE-ε4 gene. Linard and colleagues [56] reported that APOE-ε4 carriers who had signs of frequent HSV1 reactivation were also at higher risk for AD, while this connection was not seen in non-carriers of APOE-ε4. The potential influence of tailored trials based on APOE status on vaccine efficacy has yet to be explored.

## 5. Conclusions

Human alphaherpesviruses (HSV 1, HSV2, and VZV) are highly host-adapted and are responsible for widespread diseases of childhood (gingivostomatitis and chickenpox) following primary infection. Infection is lifelong, and for much of this time, the viruses maintain a dormant state (latency) in neurons of the host’s nervous system. At times, the viruses can reactivate and cause clinically apparent disease typified by shingles/zoster following VZV reactivation. Recently, zoster vaccines have become available, which have been shown to effectively prevent shingles although their efficacy declines with age. Unfortunately, clinically useful vaccines against HSV remain elusive despite interest in their development by the pharmaceutical industries. Fortunately, highly effective antiviral treatment is available against both viruses in the form of acyclovir and its analogues.

In this review, we have seen that there is increasing evidence for a role of both these viruses in the development of AD. Furthermore, interventions by vaccination or antiviral treatment may have a protective effect. Because AD has a long preclinical phase and is caused by mechanisms that are still incompletely understood, we are unable to explain how alphaherpesviruses may contribute to the development of AD. Prospective clinical trials may take many years to yield definitive answers. Much data to date have been generated through retrospective analyses of registry data with the potential for significant confounding. Added to this is the possibility that both viruses (and others) may interact to produce AD and that their effects may be different depending on host genetic factors. Other factors in need of consideration include the potential link between BCG vaccination and the prevention of HSV reactivation and how this might reduce AD risk. Further research is much needed to increase our understanding of alphaherpesviruses interactions with AD and the potential for preventive interventions through either vaccination or antiviral treatment.

## Figures and Tables

**Table 1:** The human alphaherpesviruses.

Species/ Genus/ Subfamily	Other Names	Examples of Common Clinical Conditions
*Human alphaherpesvirus* 1 *Simplexivirus* *Alphaherpesvirinae*	HSV-1—herpes simplex virus type 1; HHV-1—human herpesvirus 1.	Primary infection: herpetic skin infections (e.g., stomatitis), conjunctivitis, and encephalitis. Reactivation: cold sores. HSV-2 may also cause these.
*Human alphaherpesvirus* 2 *Simplexivirus* *Alphaherpesvirinae*	HSV-2—herpes simplex virus type 2; HHV-2—human herpesvirus 2.	Primary and recurrent genital herpes; neonatal herpes; HSV meningitis. HSV-1 may also cause these conditions.
*Human alphaherpesvirus* 3 *Varicellovirus* *Alphaherpesvirinae*	VZV—varicella zoster virus; HHV-3—human herpesvirus 3.	Primary infection: chickenpox (varicella). Reactivation: shingles (zoster).

**Table 2:** Recent studies reporting an association between HSV infection and Alzheimer’s disease.

Study	Aim	Design	Outcome
Linard M et al. [56]	To investigate the potential association of HSV infection with AD in APOE-ε4 carriers.	A prospective study of individuals who were 65 years or older, non-institutionalized, and enrolled from electoral lists; location: Bordeaux; 1258 serum samples tested.	Among HSV IgG-positive individuals, those who were IgM-positive or had a high level of IgG had a three-fold higher risk of developing AD or mixed dementia.
Shim Y et al. [57]	To investigate the association between herpesvirus infections and subsequent diagnoses of dementia.	A nationwide population-based, matched-cohort design; patients diagnosed with HSV infections aged older than 50 years; location: South Korea; 92,095 subjects diagnosed with HSV infections during 2009 followed for up to 9 years.	HSV1 infections associated with dementia. Adjusted hazard ratio for developing dementia: 1.18 (95% confidence interval: 1.16–1.200; *p* < 0.001).
Tejeda M et al. [58]	To compare the frequencies of viral species in a large sample of AD cases and controls.	An examination of whole genome and whole exome sequences derived from blood and brain samples; obtained from 37,000 participants of the Alzheimer’s Disease Sequencing Project.	Sequences processed using machine learning classifiers. Subsequent regression analyses showed that HSV1 was significantly associated with AD. Odds ratio: 3.71; *p* = 8.03 × 10^−4^.
Levine KS et al. [59]	To assess virus exposure and neurodegenerative disease risk across national biobanks.	An examination of genotyping data from Finnish and UK national biobanks to survey longitudinal and cross-sectional associations between viral exposures and neurodegenerative diseases.	Proposed strong association of viral encephalitis with dementia. In the Finnish cohort, 5.9% of viral encephalitis cases went on to develop AD compared to a general population prevalence of less than 3%.
Elhalag R et al. [60]	A systematic review and meta-analysis of HSV infection and the risk of dementia.	A comprehensive literature search with a final selection of 19 studies describing 342,535patients.	Statistically significant association between AD and increased levels of HSV IgG.

**Table 3:** Summary of treatments used for VZV infections: primary (chickenpox) and reactivation (shingles).

Treatment	Primary (Chickenpox)	Reactivation	Evidence Level
Acyclovir [68,129,130,131]	High-risk patients (immunocompromised, pregnant, etc.)	Initiated within 24 h of rash onset	Strong (RCT *s)
Immunocompromised Patients [132,133]	IV acyclovir	IV acyclovir for severe cases	Strong (guideline-based)
Valacyclovir/famciclovir [134]	Not typically used	Preferred for convenience in adults	Strong (RCTs)
Corticosteroids [135]	Not indicated	Adjunct in older adults without contraindications	Moderate (some RCTs)
Varicella zoster Immunoglobulin (VariZIG) [136]	Post-exposure prophylaxis for high-risk individuals	Not applicable	Strong (guideline-based)
Vaccination [111,137,138]	Prevention in children	For prevention of reactivation in adults	Strong (RCTs and guidelines)

* Abbreviation: RCT = randomized clinical trial.

**Table 4:** Summary of studies investigating whether herpes zoster vaccination reduces the risk of developing dementia.

Study	Aim	Design	Outcome
Scherrer J F et al. [142]	To evaluate the association between zoster vaccination and the risk of developing dementia.	A retrospective cohort study using data from the Veterans Health Administration (VHA) and MarketScan databases, encompassing over 200,000 patients.	Zoster vaccination was associated with a significantly reduced risk of dementia (VHA HR = 0.69; MarketScan HR = 0.65). The association was consistent across different age groups and races.
Eyting M et al. [143]	To assess whether herpes zoster vaccination reduces the risk of developing dementia.	The utilization of a regression discontinuity design to exploit the age-based eligibility cutoff for the zoster vaccine in Wales; the analysis of health records of 282,541 adults aged 71–88 years over a 7-year follow-up period.	Receiving the zoster vaccine reduced the probability of a new dementia diagnosis by 3.5 percentage points (a 20% relative reduction). The protective effect was more pronounced in women.
Taquet M et al. [144]	To determine if the recombinant zoster vaccine (Shingrix) is associated with a reduced risk of dementia.	A retrospective cohort study comparing individuals who received the recombinant vaccine to those who received the live vaccine, with a median follow-up of 4.15 years.	Receiving the recombinant vaccine was associated with a significantly lower risk of dementia, translating to 164 additional days lived without a dementia diagnosis among those subsequently affected.
Blandi L et al. [145]	To investigate whether herpes zoster infection increases the risk of developing dementia.	A population-based matched-cohort study analyzing health records to compare dementia incidence among individuals with and without herpes zoster infection.	The study found an increased risk of developing dementia among individuals with severe herpes zoster cases, supporting the importance of preventing and treating herpes zoster to mitigate dementia risk.

## Data Availability

Not applicable.
